# Neuropathological mRNA Expression Changes after Single Mild Traumatic Brain Injury in Pigs

**DOI:** 10.3390/biomedicines12092019

**Published:** 2024-09-04

**Authors:** Michael R. Grovola, D. Kacy Cullen

**Affiliations:** 1 Center for Neurotrauma, Neurodegeneration & Restoration, Corporal Michael J. Crescenz VA Medical Center, Philadelphia, PA 19104, USA; mgrovola@pennmedicine.upenn.edu; 2 Center for Brain Injury & Repair, University of Pennsylvania, Philadelphia, PA 19104, USA; 3 Department of Bioengineering, School of Engineering and Applied Science, University of Pennsylvania, Philadelphia, PA 19104, USA

**Keywords:** mild TBI, large animal models, mRNA, transcriptomics

## Abstract

Traumatic brain injury (TBI) is a public health concern, with an estimated 42 million cases globally every year. The majority of TBIs are mild TBIs, also known as concussion, and result from the application of mechanical forces on the head. Most patients make a complete recovery and mortality is rare; therefore, studies investigating cellular changes after mild TBI in a clinical setting are limited. To address this constraint, our group utilized a pig model of closed-head rotational acceleration-induced TBI, which recreated the biomechanical loading parameters associated with concussion on a large gyrencephalic brain similar to humans. While our previous research has focused on immunohistochemical characterization of neuropathology, the current study utilized transcriptomic assays to evaluate an array of TBI-induced neurodegenerative analytes. Pigs subjected to mild TBI were survived for 3 days post-injury (DPI) (n = 3), 30 DPI (n = 3), or 1 year post-injury (YPI) (n = 3) and compared to animals undergoing a sham procedure (n = 8). RNA was isolated from whole coronal sections of fixed tissue and multiplexed on a Nanostring neuropathology panel. Differential expression analysis revealed 11 differentially expressed genes at 3 DPI versus sham, including downregulation of the synaptotagmin calcium sensor gene (SYT1), upregulation of the neurofibromin gene (NF1), and upregulation of the Alzheimer’s disease-associated receptor gene (SORL1). There were no differentially expressed genes at 30 DPI or 1 YPI compared to shams. Additionally, high-magnitude undirected global significance scores (GSS) were detected at 3 DPI for chromatin modification and autophagy gene sets, and at 30 DPI for cytokine gene sets, while many dysregulated gene sets were highlighted by directed GSSs out to 1 YPI. This study adds to a growing body of literature on transcriptomic changes in a clinically relevant large animal model of closed-head TBI, which highlights potential therapeutic targets following mild TBI.

## 1. Introduction

Traumatic brain injury (TBI) is an extremely common injury and a major public health concern that significantly impacts morbidity and mortality around the world. There are an estimated 2.8 million reported cases of TBI in the United States and approximately 42 million cases globally every year [1,2]. However, these numbers are likely to be much higher, as the vast majority of TBIs are mild TBI, which often go unreported [2,3,4]. Mild TBI, often used interchangeably with concussion, can result from any type of mechanical force on the head such as unintentional falls or motor vehicle crashes [1]. Despite medical advances, the definition of mild TBI is still broad. Clinicians and researchers classify mild TBI in humans through an initial Glasgow Coma Scale (GCS)–an assessment and prognosis of altered consciousness–recorded in the emergency room, loss of consciousness for less than 30 min, post-traumatic amnesia of less than 24 h, and a lack of structural changes on clinical neuroimaging, such as hematoma, contusion, and brain swelling [5,6].

As most patients with mild TBI make a complete recovery and because mortality is rare, studying mild TBI in a purely clinical setting is limited [5]. Preclinical research can be used to expand our knowledge of mild TBI in a controlled environment. In particular, our research group used a pig model of closed-head rotational acceleration-induced TBI to closely emulate human TBI in numerous ways: pigs have a neuroanatomy and physiology comparable to humans, while this rotational model recreates biomechanical loading conditions ranging from mild to severe TBI and produces loss of consciousness similar to human TBI [7,8]. This model also produces the hallmark neuropathology of human TBI, diffuse axonal injury, in a similar pattern and distribution [7,9,10,11,12]. Through neuropathological analysis, our group has carefully characterized diffuse axonal injury, as well as additional neurodegenerative and neuroinflammatory changes after a single mild TBI out to 1 year post injury [13,14,15,16].

Yet, TBI is a heterogeneous injury with complex biological processes that cannot be encapsulated by immunohistochemistry alone. Advances in transcriptomic assays can provide a deeper insight into the vast array of TBI-induced degenerative, inflammatory, metabolic, and remodeling cascades by simultaneously targeting many analytes in a single sample. A few other studies have utilized genome-wide RNA sequencing after TBI in swine–one at 6 h after controlled cortical impact (CCI) TBI and a hemorrhagic shock protocol in adult pigs; another at 24 h after CCI in piglets; and another at 21 days after CCI and an electromagnetic field stimulation protocol in adult pigs–and have revealed hundreds of differentially expressed genes [17,18,19]. These studies have pioneered the examination of transcriptional response to TBI in pigs and have created critical data sources for mechanistic studies. However, these CCI studies relied on surgical craniotomies that opened the skull and exposed the brain and the surrounding dura, a pathological feature not seen in human mild TBI. Additionally, these studies did not examine the subacute and chronic timepoints when the vast majority of human mild TBI patients survive their injury. Several recent human studies have employed genome-wide analysis after mild TBI, particularly military-related or sports-related concussions [20,21,22]. While these studies identified clinically relevant biomarkers, they analyzed blood samples, which may yield complementary but distinct pathological expression profiles to brain expression profiles after TBI.

In the current study, we utilized our closed-head model of rotational acceleration-induced TBI and assessed transcriptomic expression at acute, subacute, and chronic timepoints after a single mild TBI in adult pig brains. Our previous research has shown neuropathological evidence of axonal degeneration and blood–brain barrier dysfunction at acute timepoints and morphological alterations to neuroimmune cells out to chronic timepoints [13,14,16]. Therefore, using a targeted multiplex panel of neuropathological genes, we hypothesized that various degenerative and apoptotic pathways would be upregulated at acute timepoints and that various immune-related functions would be upregulated out to chronic timepoints. This study contributes to the growing body of transcriptomic literature on clinically relevant TBI, identified key pathological pathways, and will serve as a hypothesis-generator for future TBI studies.

## 2. Materials and Methods

### 2.1. Animal Subjects

All procedures were approved by the Institutional Animal Care and Use Committees at the University of Pennsylvania and the Michael J. Crescenz Veterans Affairs Medical Center, and adhered to the guidelines set forth in the NIH Public Health Service Policy on Humane Care and Use of Laboratory Animals (2015).

For the current study, specimens were obtained from a tissue archive of castrated male pigs subjected to a single mild TBI. This tissue archive was also used in Grovola et al. [13,14,16]. All pigs were 5–6 months-old, sexually mature (considered to be young adults) Yucatan miniature pigs at a mean weight of 34 ± 4 kg (total n = 17, mean ± SD). Pigs were fasted for 16 h, then anesthesia was induced with 20 mg/kg of ketamine and 0.5 mg/kg of midazolam. Following induction, 0.1 mg/kg of glycopyrrolate was subcutaneously administered and 50 mg/kg of acetaminophen was administered per rectum. All animals were intubated with an endotracheal tube, and anesthesia was maintained with 2% isoflurane per 2 L O_2_. Heart rate, respiratory rate, arterial oxygen saturation, and temperature were continuously monitored throughout the experiment. A forced-air temperature management system was used to maintain normothermia throughout the procedure.

### 2.2. Head Rotational TBI

In order to attain closed-head diffuse mild TBI in animals, we used a previously described model of head rotational acceleration in pigs (for thorough details of our injury model, see [7,23]). Similar methods were described in Grovola et al. [13]. Briefly, each animal’s head was secured to a bite plate, which itself was attached to a pneumatic actuator and a custom assembly that converted linear motion into angular momentum. The pneumatic actuator rotated each animal’s head in the coronal plane, reaching an angular velocity between 230–270 rad/s (n = 9) (Supplemental Figure S1). Any presence of apnea was recorded (maximum apnea time = 45 s), and animals were hemodynamically stabilized if necessary. No animals were excluded from the study due to apnea or hemodynamic instability. Sham animals (n = 8) underwent identical protocols, including being secured to the bite plate; however, the pneumatic actuator was not initiated. All animals were transported back to their housing facility, monitored acutely for 3 h, and given access to food and water. Afterwards, animals were monitored daily for 3 days by veterinary staff.

### 2.3. Specimen Preparation

At 3 days post-injury (DPI) (n = 3), 30 DPI (n = 3), or 1 year post-injury (YPI) (n = 3), animals were induced and intubated as described above. Sham animals survived for 7 days (n = 3), 30 days (n = 1), or 1 year (n = 4). While under anesthesia, animals were transcardially perfused with 0.9% heparinized saline followed by 10% neutral buffered formalin (NBF). Animals were then decapitated, and tissue was stored overnight in 10% NBF at 4 °C. The following day, the brain was extracted, weighed, and post-fixed in 10% NBF at 4 °C for 1 week. To block the tissue, an initial coronal slice was made immediately rostral to the optic chiasm. The brain was then blocked into 5 mm thick coronal sections from that point by the same investigator. This allowed for consistent blocking and section coordinates across the animals. All blocks of tissue were paraffin embedded.

### 2.4. RNA Isolation and Nanostring Assay

To isolate RNA, paraffin blocks were mounted on a rotary microtome, which was cleaned with 70% ethanol and an RNase decontamination solution. Five 10 µm thick sections were obtained from a tissue block containing anterior aspects of hippocampal tissue (approximately 10 mm posterior to the optic chiasm) for each pig. Sections were placed in a 2 mL RNase-free reaction tube. Using a modified version to Roche’s High Pure FFPET RNA Isolation Kit (06650775001, Basel, Switzerland), xylene was added to the tube, which was then vortexed and briefly centrifuged, and the supernatant was removed. Absolute ethanol was added to the tube, which was then vortexed and briefly centrifuged, and the supernatant was removed. The pellet of tissue was dried and incubated overnight in a working solution of Proteinase K. The following day, the tissue was homogenized using the Miltenyi Biotech gentleMACS Dissociator (130-093-235, Bergisch Gladbach, Germany) and a fresh working solution of Proteinase K was added. Then RNA binding buffer plus absolute ethanol was added and vortexed, and the lysate was filtered through a filter tube. Next, a working solution of DNase followed by washing buffers were centrifuged through the filter fleece. Finally, we eluted RNA from the filter using an RNA elution buffer.

To assess the samples’ quantity and quality, spectrophotometry and Bioanalyzer (Agilent, Santa Clara, CA, USA) automated electrophoresis were run. We then conducted Smear Analysis from the Bioanalyzer’s electropherogram to determine each sample’s target input of 300 ng total RNA over 50 bases in length. Total RNA was multiplexed and run on an nCounter Human Neuropathology panel (Nanostring Technologies, Seattle, WA, USA) that targeted 770 genes related to themes of neurodegeneration such as neurotransmission, neuron–glia interaction, neuroplasticity, structural integrity, neuroinflammation, and metabolism. Of those 770 genes, 341 genes are over 90% homologous with pigs and were the only genes examined for the current analysis.

### 2.5. Transcriptomic Analysis

Pairwise differential expression analyses were performed with nSolver Analysis Software (version 4.0.70, Nanostring Technologies) using the fold change of RNA expression along with positive and negative controls, as well as housekeeping genes. We combined all sham specimens into one group for differential expression analysis (for additional details, see Section 3). Sham specimens were compared to each experimental condition: 3 DPI, 30 DPI, and 1 YPI. The false discovery rate was calculated via the Benjamini–Hochberg procedure. Differentially expressed genes were calculated based on log2 (fold change) compared to sham via a linear mixed model and were defined as those with an adjusted *p*-value of <0.05. Global significance scores were calculated as the square root of the mean squared t-statistic for genes in a defined gene set. Heatmaps, principal component analysis (PCA), volcano plots, and global significance scores were all generated by nSolver Analysis software. Of note, one of the enrolled specimens (a 1 year sham) was excluded from the current study due to poor quality RNA despite multiple extractions.

## 3. Results

### 3.1. Single Mild Closed-Head TBI Influences Transcriptomic Profiles

**Figure 1:**
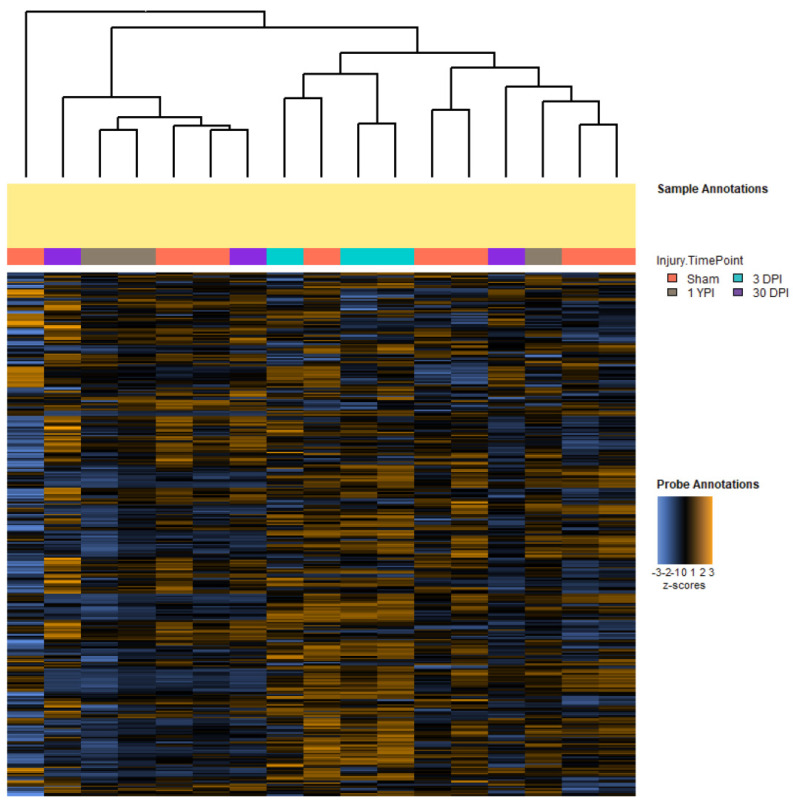
Heatmap with hierarchical clustering of all 341 genes. Specimens are represented by columns, and each gene is represented by a row. Orange indicates high expression and blue indicates low expression. Through a tree diagram, hierarchical clustering demonstrated that the three 3 DPI specimens were closely associated with each other, while specimens in other experimental groups intermingled. Additionally, there were many rows of highly expressed genes within the 3 DPI columns that may indicate gene candidates for future studies.

Our previous research has shown numerous neuropathological alterations after a single mild TBI, including neuronal hypertrophy, axonal degeneration, and neuroinflammation [13,14,16]. We thus sought to define transcriptomic changes in response to a single mild TBI using a Nanostring Neuropathology panel that evaluated genes related to neurodegeneration. We generated heatmaps with hierarchical clustering of all 341 genes as a high-level overview of our data (Figure 1). The heatmap visualized the normalized counts of the genes’ expression values, while the hierarchical clustering tree diagram helped visualize the similarities between specimens. Observationally, we saw that a small subset of genes had a QC flag, meaning that these genes did not meet the threshold requirements (greater than 50 count values across all samples) and were therefore removed from subsequent analyses. Additionally, we noted the hierarchical clustering of specimens. Specifically, the three 3 DPI specimens clustered closely together, while the other experimental groups–sham, 30 DPI, and 1 YPI–appeared to intermingle with each other in this clustering method. Moreover, the 3 DPI specimens’ columns had dozens of rows of highly expressed genes relative to the other specimens’ columns. Based on this panel of genes, we discerned that the gene expression patterns of the 3 DPI specimens were more similar to each other compared to other experimental groups, and that the rows of highly expressed genes within the 3 DPI columns may be ideal candidates for future analysis.

### 3.2. PCA Separates Transcriptomic Profiles According to Survival Timepoint

**Figure 2:**
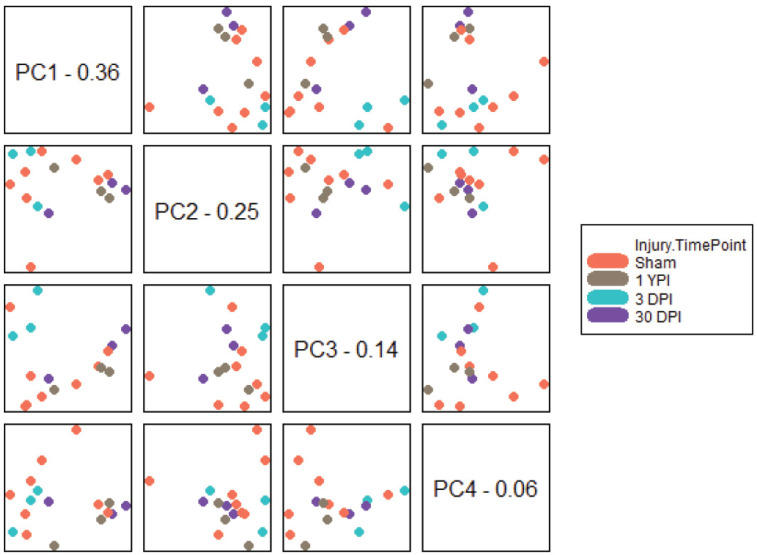
Principal component analysis of the first four principal components. Analytical data were plotted against each other, and each specimen was colored by its injury timepoint. Each PC label is followed by the percentage of variance explained by that PC. We visualized the close clustering of 3 DPI specimens along all PC1 plots, with the closest association between PC1 and PC3. The other experimental groups appeared to intermingle. However, there was relatively close clustering at PC2 versus PC4 for 30 DPI specimens, and at PC2 versus PC3 for 1 YPI specimens. These PCA results suggested that PCs can partially explain and differentiate experimental timepoint post TBI.

Similar to our heatmap, we completed PCA as a visualization technique that allows for exploratory data analysis. PCA processed our large set of transcriptomic data and transformed it into smaller sets that still contained most of the information. By reducing the dimensionality of the mild TBI specimen dataset, we had a more simplistic overview of how closely these specimens associated with their experimental group and if there was clear separation from other experimental groups. The first four PCs of the gene expression data explained 81% of the variance (Figure 2). PC1 explained 36%, PC2 explained 25%, PC3 explained 14%, and PC4 explained 6%. The first four PCs were plotted against each other and colored by each specimens’ experimental group. From here, we identified clusters in the data as well as noteworthy variables for future analysis. Broadly, we saw the 3 DPI specimens clustering relatively closely along all PC1 comparison plots, with the closest association to each other and separation from the other experimental groups between PC1 and PC3. The 30 DPI timepoint typically had two specimens clustering together, while the third specimen was more distant. The exception was the PC2 versus PC4 plot, where all 30 DPI specimens clustered closely. Finally, the 1 YPI timepoint typically had two specimens clustering closely together, while the third specimen remained more distant. The closest that all 1 YPI specimens appeared to associate was in the PC2 versus PC3 plot. Overall, our PCA results suggested that some combination of transcriptomic expression, consolidated into PCs, could partially explain and differentiate the experimental timepoint post TBI.

### 3.3. Pathway Scoring Provides a Broad Overview of Biologically Relevant Gene Sets

**Figure 3:**
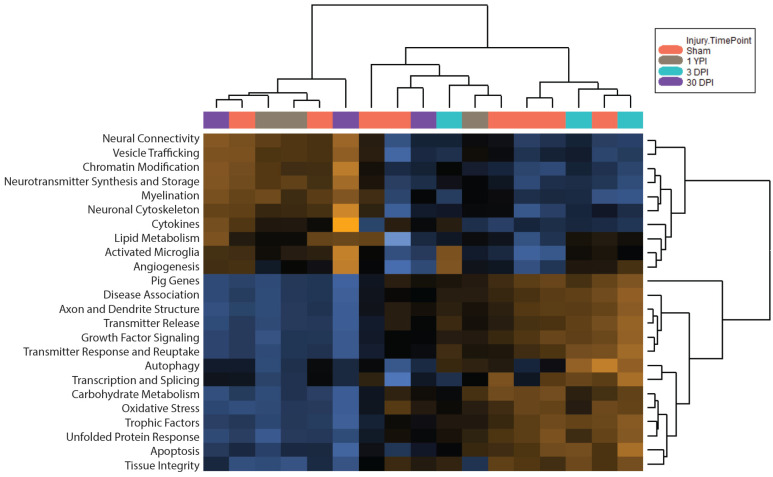
Pathway scores organized each sample’s gene expression profile into functional gene sets, where higher scores (mostly increasing expression within the gene set) are indicated in orange and lower scores (mostly decreasing expression) are indicated in blue. Each specimen is represented by a column. Pathway scores were fit using the first principal component of each gene set’s data, and tree diagrams allowed us to visualize which samples had similar profiles. At this level, we observed that two of the three 3 DPI specimens clustered closely, as well as two of the three 30 DPI specimens and two of the three 1 YPI specimens. Scores were scaled via z-transformation.

We then conducted pathway analysis to examine the attributes of the PCA. Pathway scores are an evaluation of gene expression within a functional gene set (e.g., growth factor signaling or apoptosis) consolidated from each sample’s expression profile. This high-level overview was fit using the attributes of the first principal component and oriented so that increasing scores corresponded to mostly increasing expression. Specifically, each increasing pathway score had a positive weight for at least half its genes, while, conversely, each decreasing pathway score had a negative weight for at least half its genes. A heatmap showed the change in the pathway scores across samples (Figure 3). This heatmap organized the pathway scores and clustered samples with similar profiles. We saw that two of the three 3 DPI samples clustered closely together. Similarly, two of the three 1 YPI samples clustered closely together, and two of the three 30 DPI samples clustered relatively together. Interestingly, at this level of overview, certain sham specimens clustered more closely with individual injured specimens than all three specimens of each injury group.

**Figure 4:**
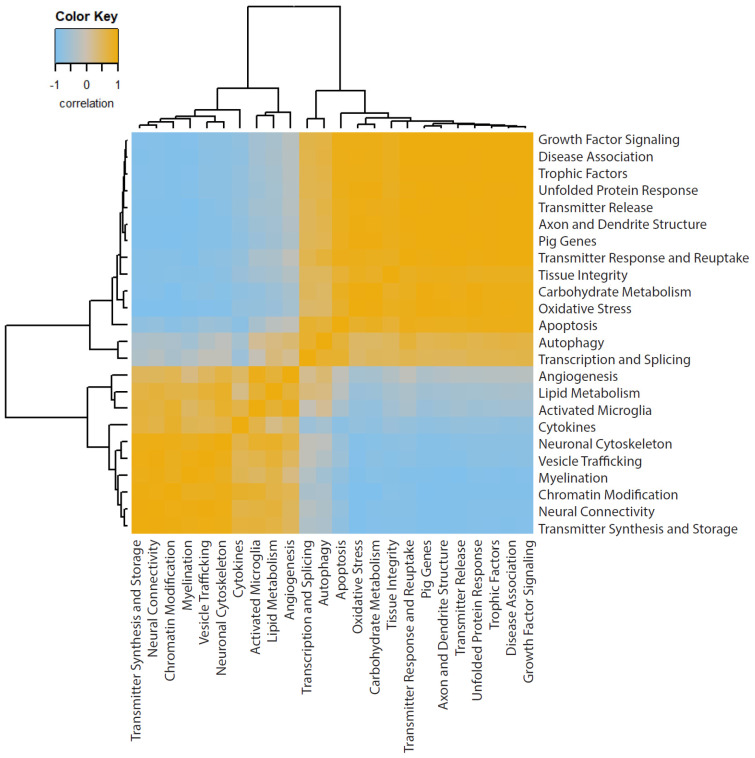
Correlation matrix heatmap of the pathway scores. Through this correlation matrix, we saw many coefficient measures, such as the strongly positive correlation of apoptosis to growth factor signaling, and the strongly negative correlation of cytokines to growth factor signaling. Orange indicates positive correlations and blue indicates negative correlations.

To supplement this pathway analysis, we also created a correlation matrix of the pathway scores (Figure 4), allowing us to indicate positive and negative correlations between two pathways and how strongly these variables related to each other. Through this correlation matrix, we saw many coefficient measures, such as the very positive correlation of apoptosis to growth factor signaling, disease association, and trophic factors. Additionally, we saw the very negative correlation of cytokines to growth factor signaling, disease association, and trophic factors. This matrix emphasized the interconnection of biologically relevant gene sets to each other in a multivariate analysis and provided insight for future mechanistic TBI studies.

### 3.4. Differential Expression Analysis Reveals Significant Transcriptomic Changes at 3 DPI

**Figure 5:**
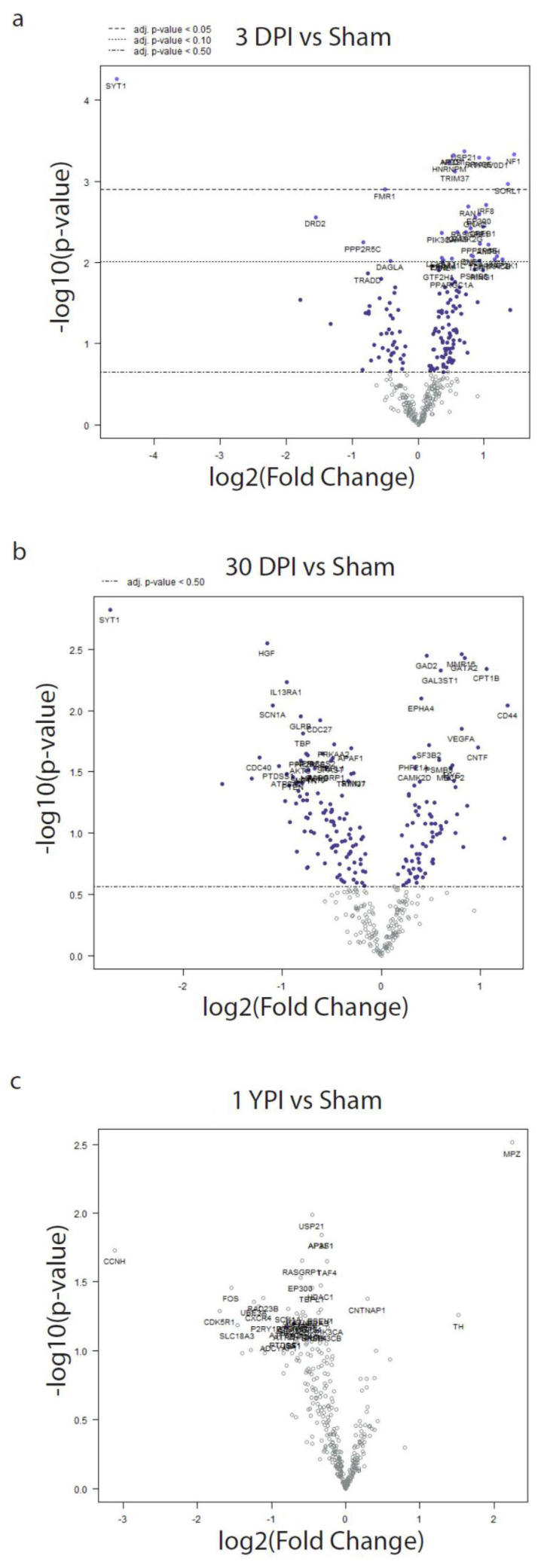
Differentially expressed genes are shown in volcano plots compared to sham. Plots depicted each gene’s −log_10_(*p*-value) and log_2_ fold change. Horizontal lines indicate false discovery rate adjusted *p*-values. Adjusted *p*-values of <0.05 are considered significant. At 3 DPI, there were 11 differentially expressed genes (**a**), while there were no differentially expressed genes at 30 DPI (**b**) and 1 YPI (**c**).

We performed differential expression (DE) analysis by normalizing mRNA expression data and then calculating statistically significant differences in the transcripts’ expression levels at each timepoint post injury. There were no significant DE genes when we compared less than 30 day sham to 1 year sham, so these animals were combined into a single sham group for analysis. Using sham specimens as the baseline reference, 11 genes were differentially expressed at 3 DPI (Figure 5). Several highly differentially expressed genes included the synaptotagmin calcium sensor gene *SYT1* that was significantly downregulated (adj. *p* = 0.0192), and the disease-associated sorting receptor gene *SORL1* (adj. *p* = 0.0375) and the neurofibromin gene *NF1* (adj. *p* = 0.0253), which were both significantly upregulated. Table 1 presents all the significant and other notable DE genes at 3 DPI. There were no statistically significant DE genes at the 30 DPI and 1 YPI timepoints.

### 3.5. Global Significance Scores Summarize the DE Analysis by Gene Set

**Figure 6:**
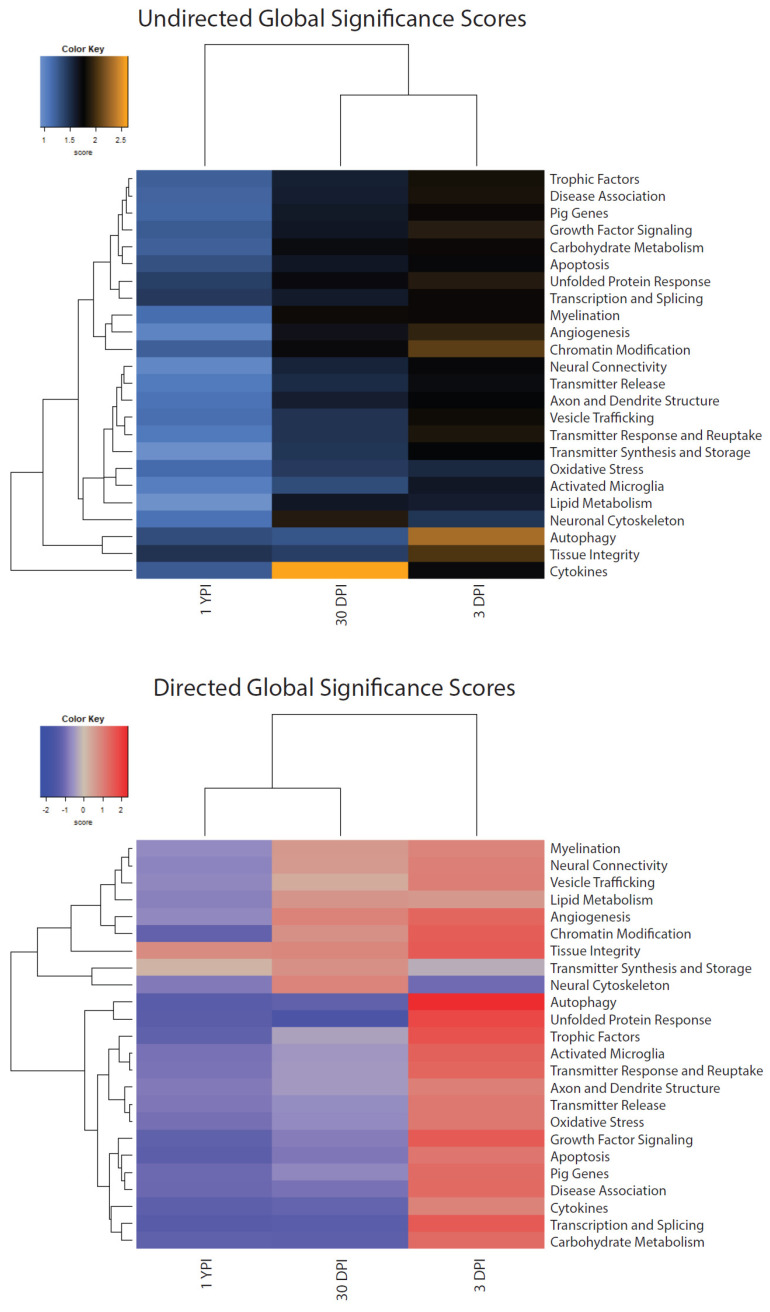
Heatmap displaying each injury timepoint’s undirected and directed global significance score (GSS). Each gene set’s most differentially expressed genes were summarized, and the extent was scored using a GSS. For undirected GSSs, orange indicates extensive differential expression and blue indicates less differential expression. For directed GSSs, red indicates upregulation of the gene set’s genes and blue indicates downregulation.

The results of DE analysis were summarized at a gene set level and the extent of expression was indicated by a global significance score (GSS). A GSS is essentially a summary of the DE analysis t-statistics from all genes in each gene set, with scores of greater magnitude indicating a stronger pattern of gene set changes. Additionally, these scores were scaled to the same distribution so we could make comparisons between different pathways. We used a heatmap to display each experimental group’s undirected and directed GSS (Figure 6). An undirected GSS measures the extent of DE of the genes in a particular gene set, ignoring whether a gene is upregulated or downregulated within a set. In contrast, a directed GSS measures the extent of upregulation or downregulation of genes within a gene set. There were particularly high undirected GSSs for chromatin modification and autophagy gene sets at 3 DPI compared to sham, as well as an exceptionally high undirected GSS for cytokines at 30 DPI compared to sham. Among the upregulated directed GSSs at 3 DPI were gene sets related to activated microglia, autophagy, chromatin modification, growth factor signaling, tissue integrity, transcription and splicing, trophic factors, and unfolded protein response, while neuronal cytoskeleton genes were downregulated. At 30 DPI, gene sets related to autophagy, carbohydrate metabolism, cytokines, transcription and splicing, and unfolded protein response had downregulated directed GSSs compared to sham. Finally at 1 YPI, a number of gene sets had downregulated directed GSSs including apoptosis, autophagy, cytokines, and unfolded protein response. Interestingly, tissue integrity genes had an upregulated directed GSS at 1 YPI compared to sham. Table 2 displays each experimental group’s undirected and directed GSS.

## 4. Discussion

For the current study, we hypothesized that degenerative and apoptotic pathways would be upregulated acutely, and that immune-related functions would be upregulated out to chronic timepoints. Using a panel of 341 genes that are highly relevant to TBI neuropathology, we identified 11 differentially expressed genes at 3 DPI after a single mild closed-head rotational acceleration-induced TBI in pigs. These DE genes have a wide range of functions such as synaptic connectivity, neuron structure, disease association, and cell proliferation. Additionally, GSS calculations revealed high-magnitude expression changes: there were high undirected GSSs at 3 DPI for chromatin modification and autophagy gene sets and at 30 DPI for cytokine gene sets, while many dysregulated gene sets were highlighted by directed GSSs out to 1 YPI. There were no DE genes detected at 30 DPI or 1 YPI, so we were compelled to partially reject our hypothesis.

To our knowledge, only three other studies have conducted transcriptomic analysis in a pig model of TBI. First, Shin et al. investigated gene expression changes in the ipsilateral versus contralateral sides of cerebral cortex after CCI in eight female piglets; this study did not include a sham injury group [17]. At 24 h, animals were euthanized for RNA sequencing, and 6642 DE genes were identified between the ipsilateral and contralateral sides, with many of the highly upregulated DE genes related to immune response such as CD4, CD86, and several interleukin-related genes. In another study, Nikolian et al. subjected 10 female pigs to CCI brain injury and a hemorrhagic shock protocol [18]. Then, pigs were either treated with valproic acid or normal saline; this study did not include a sham injury group or a CCI injury alone group. Six hours after injury, the animals were euthanized for RNA sequencing. Valproic acid treatment upregulated genes involved in nervous system morphology, neuronal development, and neuron quantity, while genes related to apoptosis, glial proliferation, and neuroepithelial cell differentiation were downregulated. Finally, in a recent study, Rai et al. conducted CCI (3.579 m/s) and electromagnetic field stimulation on three male Yucatan minipigs that were survived to 21 DPI. One animal received EMF measurements the same day as the injury, one animal was measured 2 days after injury, and one animal was without EMF measurements, which resulted in n = 1 for each experimental category. This study did not include a sham group; tissue was collected from the injured ipsilateral cortex and compared to the contralateral hemisphere for RNA sequencing, which revealed 175 DE genes [19]. Unlike the previously mentioned pig-based TBI transcriptomic studies, our study used a closed-head model of TBI, which is critical for modeling clinically defined mild TBI in humans. Furthermore, we used a targeted panel of genes with a limited number of transcripts, while RNA sequencing allowed the full transcriptome to be reviewed. Considering the limited existing literature on TBI transcriptomics utilizing a large animal model, we contend that this study offers crucial insights to this expanding subfield of TBI research.

Our detected DE genes at 3 DPI are involved in a variety of biological processes. The most significantly downregulated gene, SYT1 (−4.57-fold), encodes for synaptotagmin-1, a calcium-sensing synaptic vesicle protein that regulates exocytosis of neurotransmitters [24]. Nishiki and Augustine demonstrated that synaptotagmin-1 synchronizes neurotransmitter release in mouse hippocampal neurons [55]. Furthermore, Hussain et al. found that both presynaptic and postsynaptic synaptotagmin-1 concentrations were reduced in a rat model of temporal lobe epilepsy compared to control animals [25]. They suggested that this downregulation is an adaptive measure to decrease calcium sensitivity in excitotoxic conditions. Excitotoxicity and post-traumatic epilepsy are increasingly examined in human TBI studies, indicating potential long-term neurological deficits even in cases of mild TBI [56,57]. Wolf et al. demonstrated synaptic dysfunction and hippocampal hyperexcitability after a single mild TBI using the current injury model [58]. Therefore, it is plausible that SYT1 may be downregulated in response to TBI-induced hyperexcitability; however, further investigations using electrophysiological and protein-level analyses will be needed.

At 3 DPI, NF1 (1.45-fold) was the most significantly upregulated DE gene. NF1, a tumor suppressor gene, encodes for neurofibromin, a protein involved in numerous cell signaling pathways, acting as a regulator of cell proliferation and migration, neurite outgrowth, and cytoskeleton support [59]. In a recent study, Chatterjee et al. explored the interconnection of NF1 and TBI by subjecting nf1 mutant mice to optic nerve crush injury as well as CHIMERA TBI [28]. Optic gliomas formed after both types of injury in mice with nf1-deficient preneoplastic inhibitors, suggesting that NF1 may play a protective role in TBI.

Several other DE genes are related to cell proliferation or cell death. TRIM37 (0.558-fold) encodes for an E3 ubiquitin ligase that inhibits the transcription of several genes. TRIM37 is upregulated in glioma samples, and suppression of TRIM37 inhibited glioma proliferation and migration, and induced apoptosis [41]. Han et al. found that intracerebral hemorrhage-induced thrombin expression upregulated TRIM37, which induced apoptosis and IL-1β release from microglia [42]. Interestingly, our previous research found an increase in fibrinogen, a blood protein and indicator of blood–brain-barrier disruption, and homeostatic changes to microglia in the brain parenchyma at 3 DPI [16]. PRKCE (0.92-fold) encodes protein kinase C epsilon and has been shown to induce astrocytic differentiation of neural progenitor cells, and has been explored as a therapeutic strategy to regenerate injured brains [34,35]. Activation of the epsilon form of protein kinase C is involved in the protective mechanism of ischemic preconditioning, a protective mechanism where the tissue is resistant to damage following subthreshold ischemic events [60]. EP300 encodes for a histone acetyltransferase and regulates neurogenesis. Moreover, ep300 has been shown to decrease following stab injury in a zebrafish model [51].

Several DE genes are disease associated. SORL1 (1.36-fold) encodes for a sorting protein-related receptor that is downregulated in Alzheimer’s disease (AD) and is implicated in the trafficking and processing of amyloid precursor protein (APP) [43]. APP accumulation in damaged axons is the hallmark pathology of TBI and has been thoroughly characterized in our pig model of TBI [12,14,61,62]. To assess sorl1 expression after TBI, Lamprecht et al. employed two injury models: moderate mechanical stretch injury to hippocampal slice cultures and moderate CCI in rat pups [44]. The expression of sorl1 was downregulated in both immunohistochemistry and Western blotting, implicating sorl1 in TBI pathology and providing another link between TBI and AD [63]. Also related to AD pathology, ATP6V0D1 (1.07-fold) encodes for a vacuolar ATPase that mediates the acidification of intracellular organelles and is thought to have a neuroprotective function in AD [36]. Using a rodent FPI model of moderate TBI, Meng et al. found that TBI affected the alternative splicing of ATP6V0D1 [37]. Finally, RAN encodes for a Ras-related nuclear protein and is involved in transport, the cell cycle, and proliferation. Low expression levels of RAN resulted in transport disruption between the nucleus and cytoplasm in AD brains, while higher RAN expression protected against hippocampal neuron death in vitro [49,50].

We detected two DE genes that are involved in immune function and response. USP21 (0.698-fold) encodes for a ubiquitin-specific peptidase that is involved in many biological processes. Molecular studies have demonstrated that USP21 inhibits tumor necrosis factor alpha-induced nuclear factor kappa B activation, which are known innate immune system-associated genes [26]. USP21 also regulates protein stability and stem cell pluripotency, is dysregulated in models of stroke, and exerts oncogenic functions [27,64,65]. In addition, IRF8 (1.03-fold) is a critical regulator of microglial reactivity [47]. Using an optic nerve crush injury model, Zhou et al. noted IRF8′s synergistic binding with PU.1 proteins in the process of microglial activation that cooperatively targeted many microglia activation-related genes, such as NF-kappaB pathway signals [48]. Our previous research described homeostatic changes as well as density changes to microglia in certain neuroanatomical regions [13,14,16] and, paired with the current study, indicated that IRF8 may be a novel target for control of microglia under neurodegenerative conditions. Moreover, while we did not detect DE genes at 30 DPI, cytokines had the highest undirected global significance score and a moderately downregulated directed global significance score. This scoring mismatch suggests cytokine-related gene dysregulation at 30 DPI and merits further analysis.

We have identified several DE genes related to cell structure. AP3S1 (0.53-fold) encodes for a subunit of an adapter complex involved in subcellular protein trafficking and Golgi body vesicle budding. Using a lateral fluid percussion model of TBI in rats, Boone et al. suppressed a set of 600 genes with microRNA to determine their biological role after injury [29]. Among these genes, ap3s1 was identified as essential for neuronal function. NEO1 (0.522-fold) encodes for neogenin, a multifunctional cell surface receptor. Within the central nervous system, NEO1 mediates the action of repulsive guidance molecules, which are implicated in axonal guidance and neural tube closure [30]. NEO1 is expressed in neural stem cells in the subventricular zone and hippocampal subgranular zone, where it promotes cell genesis [31,32]. Sun et al. demonstrated that NEO1 is decreased in the hippocampi of human epilepsy patients [33]. Additionally, using a knock-out model of neo1, Sun et al. showed that knocking out neo1 in hippocampal astrocytes, but not neurons, increased epileptiform spikes and therefore NEO1 may protect the brain from epilepsy.

Interestingly, several DE genes are related to neural connectivity or synaptic integrity. Depletion of HNRNPM (0.472-fold), which encodes an RNA-binding protein, resulted in cognitive decline, the loss of synaptic proteins, and alterations in dendritic spine density [38]. Furthermore, HNRNPM interacts with other binding proteins in biological processes that are important for neuronal health, while the larger family of HNRNPs have been implicated in frontotemporal lobar degeneration and amyotrophic lateral sclerosis [39,40]. GNAQ encodes a guanine nucleotide-binding protein subunit, and activation of GNAQ upregulates synaptic plasticity and improves memory [54]. DRD2 encodes for a dopamine D2 receptor, and variants of this receptor influence cognitive recovery following TBI [52,53]. Finally, FMR1 (−0.501-fold), whose gene mutation is typically associated with Fragile X syndrome, was found to be highly expressed in the brain and may have a function in germ cell proliferation [66]. It encodes for an RNA-binding protein that regulates the transport and translation of many mRNAs in the brain, and loss of this protein results in altered synaptic function and plasticity [45,46].

Our higher-level analyses–heatmaps with hierarchical clustering, PCA, pathway analysis, and GSS–demonstrated that 3 DPI specimens were generally associated with each other, while the other experimental groups generally intermingled. This suggests that, based on the RNA panel used, 3 DPI specimens had a more distinct RNA expression profile compared to sham, and that 30 DPI and 1 YPI specimens had expression profiles which more closely resembled sham expression profiles. PCA results appeared to partially differentiate between all experimental timepoints, but the 3 DPI specimens remained the most observationally distinct with their clustering.

Both our pathway analysis and GSS allowed us to examine expression changes in sets of genes, yet they differed in distinct ways. Pathway scores were generated for individual samples based on the first principal component of the PCA, which, in our study, accounted for only 36% of the variance in the data and was given a weighted expression level. Therefore, changes may be detected from only a small number of genes in a gene set. GSSs were generated from DE test statistics and presented at the experimental group level. All genes in a gene set were given equal weight in the GSS, and, therefore, more genes in a gene set needed to be dysregulated to result in a large magnitude GSS. One example to illustrate these differences is the cytokine gene set at 30 DPI. We noted an exceptionally high-magnitude undirected GSS, but not directed GSS, for the cytokine gene set at 30 DPI, which suggests dysregulation of cytokine gene expression that was not predominately upregulated or downregulated. Through our pathway analysis (which only explained 36% of the variance), we also saw that two 30 DPI specimens had an increased expression score, while the third 30 DPI specimen was close to baseline expression. GSS and pathway analysis scores alone presented an incomplete picture of cytokine activity at 30 DPI, but these high-magnitude changes suggested that further examination of cytokine activity at this timepoint are warranted.

This study has several limitations. As previously described, we used a targeted panel of neuropathological related genes, unlike other studies that examined genome-wide alterations using RNA sequencing. Moreover, this panel of genes was further limited by gene homology: only 341 genes out of 770 were deemed sufficiently homologous to pigs. While future transcriptomic analysis in pig TBI tissue should apply this genome-wide approach, there are fewer identified genes in pigs compared to humans and rodents. Therefore, implications will remain limited until repeated analyses have been conducted to ascertain the full impact and relevance of DE genes in pigs. Additionally, RNA was isolated from whole coronal sections of fixed tissue, where small, region-specific transcriptomic changes may be overlooked. Future analyses should include in situ hybridization that targets not only DE genes but also genes that narrowly missed our significance cutoff, genes within gene sets with high global significance scores, and genes related to protein-level immunohistochemical pathology. Additionally, integrating proteomic analyses would permit a more comprehensive understanding of the molecular expression and allow us to verify the protein abundance of our DE genes.

Overall, the current study adds to a small yet growing body of literature on transcriptomic changes in a clinically relevant large animal model of closed-head TBI. We identified numerous DE genes at 3 DPI, but not at 30 DPI or 1 YPI, after a single mild TBI in pigs. This data may detect potential therapeutic targets but also serve as a hypothesis generator for future TBI studies, with the ultimate goal of providing treatment to human TBI survivors.

## Figures and Tables

**Table 1:** Differentially expressed genes at 3 DPI compared to sham.

Gene	Fold Change (log2)	Lower 95% Confidence Limit (log2)	Upper 95% Confidence Limit (log2)	Adjusted *p*-Value	Biological Function and Role
*SYT1*	−4.57	−6.09	−3.04	0.0192	Calcium-sensing synaptic vesicle protein [24] Regulates neurotransmitter release Downregulated in epilepsy models [25]
*USP21*	0.698	0.407	0.99	0.0253	Ubiquitin-specific peptidase Innate immune system regulator [26] Dysregulated in stroke models [27]
*NF1*	1.45	0.838	2.07	0.0253	Neurofibromin Potentially protective role in TBI [28]
*AP3S1*	0.53	0.306	0.755	0.0253	Adapter complex involved in subcellular protein trafficking Essential for neuronal function [29]
*NEO1*	0.522	0.300	0.743	0.0253	Axonal guidance and neural tube closure [30] Promotes cell genesis in the subventricular zone and hippocampal subgranular zone [31,32] Protective in epilepsy [33]
*PRKCE*	0.92	0.527	1.31	0.0253	Protein kinase C epsilon Induces astrocytic differentiation [34,35]
*ATP6V0D1*	1.07	0.610	1.52	0.0253	Vacuolar ATPase Potentially neuroprotective in AD [36] Alternative splicing affected by moderate FPI [37]
*HNRNPM*	0.472	0.267	0.677	0.0253	RNA-binding protein Depletion related to cognitive decline [38,39,40]
*TRIM37*	0.558	0.308	0.808	0.0292	E3 ubiquitin ligase Role in apoptosis [41,42]
*SORL1*	1.36	0.723	2.00	0.0375	Sorting protein-related receptor Trafficking and processing APP [43] Downregulated in AD and after moderate CCI [43,44]
*FMR1*	−0.501	−0.74	−0.261	0.0396	Possible role in germ cell proliferation Protein loss alters synaptic function and plasticity [45,46]
*IRF8*	1.03	0.510	1.56	**0.0547**	Regulator of microglial reactivity [47,48]
*RAN*	0.765	0.375	1.15	**0.0547**	Ras-related nuclear protein Cell cycle and cell proliferation Downregulated in AD [49] Higher expression is neuroprotective [50]
*EP300*	0.924	0.438	1.41	**0.0610**	Histone acetyltransferase Regulates neurogenesis Downregulated in a stab injury model [51]
*DRD2*	−1.55	−2.37	−0.723	**0.0610**	Dopamine receptor Influences cognitive recovery following TBI [52,53]
*GNAQ*	0.865	0.404	1.33	**0.0610**	Upregulation involved in synaptic plasticity and memory [54]

**Table 2:** The undirected and directed global significance scores (GSS) for each injury timepoint compared to sham.

Gene Set	Undirected 3 DPI	Undirected 30 DPI	Undirected 1 YPI	Directed 3 DPI	Directed 30 DPI	Directed 1 YPI
Activated Microglia	1.642	1.333	1.043	1.463	−0.492	−0.937
Angiogenesis	1.939	1.679	1.013	1.41	0.968	−0.682
Apoptosis	1.749	1.649	1.31	1.173	−0.871	−1.255
Autophagy	2.32	1.287	1.329	2.32	−1.204	−1.316
Axon and Dendrite Structure	1.764	1.6	1.116	1.028	−0.461	−0.864
Carbohydrate Metabolism	1.791	1.715	1.233	1.307	−1.216	−1.179
Chromatin Modification	2.081	1.734	1.234	1.558	0.74	−1.17
Cytokines	1.734	2.618	1.254	0.966	−1.138	−1.233
Disease Association	1.843	1.594	1.204	1.331	−0.93	−1.051
Growth Factor Signaling	1.899	1.649	1.248	1.613	−0.801	−1.186
Lipid Metabolism	1.61	1.641	0.922	0.632	0.678	−0.768
Myelination	1.79	1.802	1.147	0.931	0.618	−0.669
Neural Connectivity	1.749	1.566	0.997	1.015	0.583	−0.726
Neuronal Cytoskeleton	1.47	1.89	1.125	−1.006	0.954	−0.87
Oxidative Stress	1.544	1.445	1.165	1.114	−0.664	−0.946
Pig Genes	1.796	1.626	1.2	1.309	−0.697	−1.018
Tissue Integrity	2.033	1.414	1.481	1.606	0.898	0.832
Transcription and Splicing	1.793	1.619	1.447	1.61	−1.287	−1.333
Transmitter Release	1.711	1.522	1.068	1.112	−0.633	−0.872
Transmitter Response and Reuptake	1.853	1.479	1.076	1.406	−0.46	−0.915
Transmitter Synthesis and Storage	1.776	1.466	0.929	−0.249	0.739	0.173
Trophic Factors	1.834	1.589	1.238	1.724	−0.377	−1.179
Unfolded Protein Response	1.894	1.726	1.412	1.857	−1.568	−1.287
Vesicle Trafficking	1.804	1.483	1.144	1.041	0.31	−0.699

## Data Availability

The datasets used during the current study are available from the corresponding author upon reasonable request.

## Supplementary Materials

The following supporting information can be downloaded at: https://www.mdpi.com/article/10.3390/biomedicines12092019/s1. Figure S1: Methodology for closed-head rotational acceleration TBI in swine. Diffuse brain injury was induced using rotational acceleration–deceleration of the head/brain in the coronal plane. Ref. [7] is cited in the Supplementary Materials.
