# Supplementary material for: Neuropathological mRNA Expression Changes after Single Mild Traumatic Brain Injury in Pigs
Source: Biomedicines. 2024 Sep 4;12(9):2019. doi: 10.3390/biomedicines12092019 (PMC11428889; doi:10.3390/biomedicines12092019)
Supplement: Supplementary file 1 [file biomedicines-12-02019-s001.zip › biomedicines-3136560-supplementary.pdf]

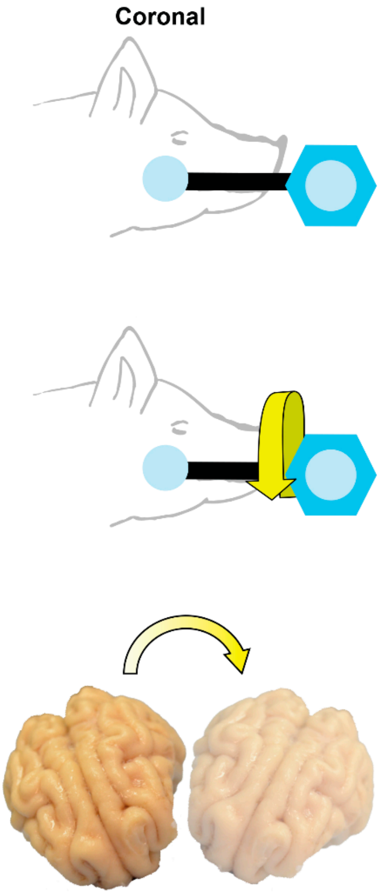

Supplemental Figure S1. Methodology for closed-head rotational acceleration TBI in swine. Diffuse brain injury was induced using rotational acceleration–deceleration of the head/brain in the coronal plane. Adapted from (7).
